# Dissection of Influenza A Virus M1 Protein: pH-Dependent Oligomerization of N-Terminal Domain and Dimerization of C-Terminal Domain

**DOI:** 10.1371/journal.pone.0037786

**Published:** 2012-05-24

**Authors:** Ke Zhang, Zhao Wang, Xiaoling Liu, Changcheng Yin, Zeshan Basit, Bin Xia, Wenjun Liu

**Affiliations:** 1 Center for Molecular Virology, CAS Key Laboratory of Pathogenic Microbiology and Immunology, Institute of Microbiology, Chinese Academy of Sciences, Beijing, China; 2 Graduate University of Chinese Academy of Sciences, Beijing, China; 3 Department of Biophysics, Health Science Center, Peking University, Beijing, China; 4 Beijing Nuclear Magnetic Resonance Center, Peking University, Beijing, China; University of Hong Kong, Hong Kong

## Abstract

**Background:**

The matrix 1 (M1) protein of Influenza A virus plays many critical roles throughout the virus life cycle. The oligomerization of M1 is essential for the formation of the viral matrix layer during the assembly and budding process.

**Methodology/Principal Findings:**

In the present study, we report that M1 can oligomerize *in vitro*, and that the oligomerization is pH-dependent. The N-terminal domain of M1 alone exists as multiple-order oligomers at pH 7.4, and the C-terminal domain alone forms an exclusively stable dimer. As a result, intact M1 can display different forms of oligomers and dimer is the smallest oligomerization state, at neutral pH. At pH 5.0, oligomers of the N-terminal domain completely dissociate into monomers, while the C-terminal domain remains in dimeric form. As a result, oligomers of intact M1 dissociate into a stable dimer at acidic pH.

**Conclusions/Significance:**

Oligomerization of M1 involves both the N- and C-terminal domains. The N-terminal domain determines the pH-dependent oligomerization characteristic, and C-terminal domain forms a stable dimer, which contributes to the dimerization of M1. The present study will help to unveil the mechanisms of influenza A virus assembly and uncoating process.

## Introduction

Influenza viruses are enveloped viruses that belong to the *Orthomyxovirus* family. The genome of influenza A virus contains eight negative-stranded RNA segments, which code for 10 or 11 proteins [1], [2], [3], [4], [5]. The matrix 1 (M1) protein, encoded by segment 7, is the most abundant structural protein in the virions [6], [7]. M1 is a multifunctional protein that plays essential roles in many aspects of the virus life cycle. It forms the matrix layer localized between the lipid membrane and the viral ribonucleoproteins (vRNPs) [8], ensuring the stabilization of the architecture of the virion. By interacting with RNA and vRNPs [9], [10], [11], M1 is also involved in nuclear localization [12], RNA transcription inhibition [13], [14], [15], and regulation of the import/export of newly synthesized vRNPs [16], [17], [18]. As the major structural protein, M1 plays an important role in virus assembly and budding. It can form virus-like particles (VLPs) through collaboration with other viral proteins [19], [20], [21], [22]. During budding, M1 brings viral components to the budding site [23], interacts with viral envelope proteins (HA, NA, M2) [24], [25], [26], and also recruits host components needed for bud completion [24], [27], [28].

M1 protein is made up of 252 amino acid residues [29], and consists of two domains (N-terminal domain from 1 to 164 aa and C-terminal domain from 165 to 252 aa) linked by a protease-sensitive loop. The three-dimensional structure of the N-terminal domain was determined by X-ray diffraction at pH 4.0 and pH 7.0 [30], [31], [32]. The structures showed that the N-terminal domain consists of two 4-helix bundles (2 to 67 aa and 91 to 158 aa) connected by a helix linker (H5). The three dimensional structure of C-terminal domain has not been obtained so far, but data from circular dichroism (CD), tritium bombardment and bioinformatics analysis suggest that C-terminal domain folds into helices and contains an appreciably unstructured region [30], [33], [34], [35], [36].

Previous investigations have also shown that M1 has a strong tendency to oligomerize [37]. In virus assembly and the budding, the oligomerization of M1 is required for the matrix layer to form under the lipid membrane [21], [38], [39], [40], [41]. Furthermore, the M1-M1 interaction facilitates membrane bending, which is required for bud initiation [38], [41]. Expression of M1 alone in eukaryotic cells allows for the production of VLPs [21], [22]. While in authentic virions, M1 forms an ordered structure adjacent to the envelope [42]. It has been reported that the N-terminal domain mediates the oligomerization of M1. The crystal structure of the N-terminal domain showed that it dimerizes through the interaction interfaces [31], [32], and that the 91–158 aa region is the main determinant of M1 self-oligomerization [10].

Interestingly, Noton *et al.* reported that the C-terminal domain also plays an important role in oligomerization by interacting with the N-terminal domain, but not with other C-terminal domains [10]. Furthermore, Ruigrok *et al.* found that the C-terminal domain is involved in M1 oligomerization, for the C-terminal alone could cause aggregation [43]. Studies of the behavior of the C-terminal domain have produced conflicting results, which lead to future investigation into fully elucidating the role of C-terminal domain in M1 oligomerization.

During the uncoating process of virus infection, the virion is acidified by the influx of H^+^ [44], [45], [46]. A structural transition of the matrix layer has been observed when the virus was incubated at low pH [42]. Recent research by cryo-electron tomography further showed that the intermolecular interactions in the M1 layer are affected when the virions were incubated at pH 4.9, and the matrix layer was no longer seen in the virions [47]. The interaction between M1 and vRNP has also been shown to be disrupted by low pH [9]. Zhirnov found that M1 extracted from M1-vRNP complexes at an acidic pH is in a monomeric form and does not aggregate after pH neutralization [9], [15]. But the crystal structure of the N-terminal domain solved by Harris at pH 4.0 suggested that this domain is a dimer [32]. Therefore, it is not certain which oligomerization state of M1 forms in acidic pH, and how the influence of pH affects the oligomerization of M1.

In order to resolve these aforementioned issues, we investigated the oligomerization of M1 and determined the individual contribution of the N- and C-terminal domains. We found that the oligomerization of M1 is pH-dependent. M1 can form multiple-ordered oligomers at neutral pH, and those oligomers dissociate at acidic pH to dimeric form. Further studies revealed that pH-dependent oligomerization characteristic of M1 is due to the N-terminal domain. The C-terminal domain exists as a stable dimer in solution, independent of pH and concentration.

## Results

### The oligomerization of M1 is dependent on pH

It has been reported that M1 forms an organized structure adjacent to the envelope in virus particles [42]. Ruigrok *et al.* extracted M1 oligomers from influenza A virus [41] and Zhao *et al.* reported that M1 tends to oligomerize soon after synthesis in BHK-21 cells [48]. To investigate the oligomerization of M1 *in vitro*, we constructed recombinant M1 with a C-terminal His_6_-tag, which was produced in soluble form in *E. coli*. M1 was purified by nickel affinity chromatography and showed a single band at about 28 kDa on SDS-PAGE (Fig. S1A, lane 3). CD spectroscopy analysis showed that purified M1 was a typical α-helical protein (Fig. S1B). Consistent with the previous report [32], the result showed that recombinant M1 was not stable and that the protein could degrade

**Figure 1:**
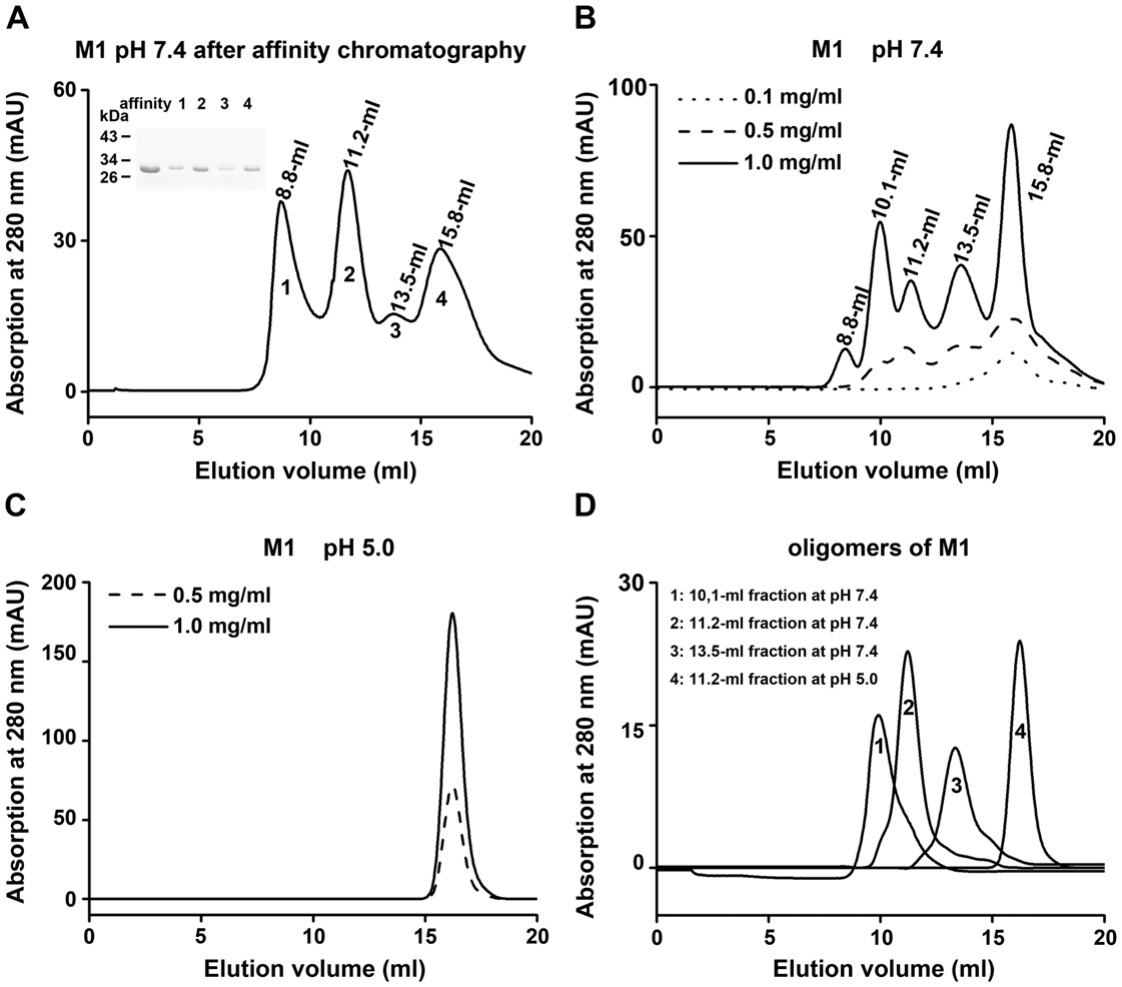
The oligomerization of M1 analyzed by gel filtration. The oligomerization of M1 was analyzed on a Superdex 200 HR 10/30 column. (A) Purified M1 from nickel affinity chromatography yielded four peaks, whose elution volumes are indicated. The samples from each peak were collected and analyzed by SDS-PAGE. (B) The 15.8-ml fraction (from panel A) was collected, concentrated and re-applied on the gel filtration column at different concentrations, at pH 7.4. (C) Different concentrations of M1 all eluted as a single peak on the gel filtration column at pH 5.0. (D) Peak 1, 2, 3 correspond to the 10.1-ml, 11.2-ml, and 13.5-ml fractions of M1 re-applied on the gel filtration column at pH 7.4, respectively. Peak 4 corresponds to the 11.2-ml fraction re-applied on the gel filtration column at pH 5.0.

**Figure 2:**
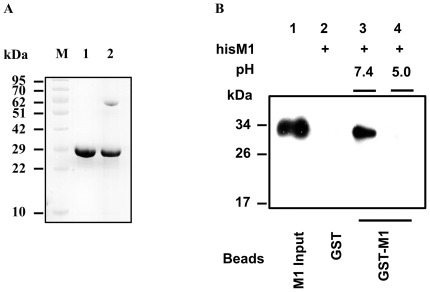
Cross-linking assay of M1 and GST pull-down assay detected the direct interaction between M1 molecules. (A) Lane 1 is M1 in the absence of cross-linker, and lane 2 is the cross-linked M1 dimer. (B) Glutathione-Sepharose bound GST-M1 or GST was incubated with hisM1 in the binding buffer (pH 7.4). After washing extensively with the buffer of different pH (7.4 or 5.0), the hisM1 bound to the beads was extracted and analyzed by immunoblotting with anti-His antibody.

Purified M1 from nickel affinity chromatography at a concentration of 0.8 mg/ml was eluted from the gel filtration column in four major fractions at pH 7.4, whose elution volumes were 8.8 ml, 11.2 ml, 13.5 ml, and 15.8 ml, respectively (Fig. 1A). The apparent molecular mass of the 15.8-ml fraction was estimated to be 52 kDa, approximately double of that of the M1 monomer (28.9 kDa) (Fig. S2). The result indicated that the smallest oligomerization state of M1 is a dimer. The oligomerization state of 15.8-ml fraction of M1 was further confirmed by cross linking assay. After treating with glutaraldehyde, the sample showed two bands on Tricine-SDS-PAGE. One band was at the same position with the untreated sample (28 kDa), while the other ran to the position of about 56 kDa, which is the molecular mass of an M1 dimer (Fig. 2A). The dimeric M1 eluted from a gel filtration column was collected and concentrated to different concentrations by Ultra filtration, and then re-applied to the gel filtration column at pH 7.4. In this process, M1 exhibited concentration-dependent oligomerization (Fig. 1B). When the loaded protein reached a concentration of 0.1 mg/ml, M1 was eluted from the gel filtration column as a single fraction of 15.8 ml. However, with an increase of protein concentration to 0.5 mg/ml, M1 yielded four fractions with the elution volumes of 10.1 ml, 11.2 ml, 13.5 ml, and 15.8 ml, corresponding to the molecular masses of 580 kDa, 460 kDa, 155 kDa, and 52 kDa, respectively. An 8.8-ml fraction was observed on the gel filtration column when the loaded protein concentration reached 1.0 mg/ml, indicating M1 is able to form higher molecular mass oligomers which beyond the detection limits of the column. The proportion of 10.1-ml, 11.2-ml, and 13.5-ml fractions also increased with increasing of sample concentration. We further collected 10.1-ml, 11.2-ml, and 13.5-ml fractions, respectively, and re-applied the samples to the gel filtration column at pH 7.4. The proteins continued to elute at the same volumes (Fig. 1D). Even if the 10.1-ml, 11.2-ml, and 13.5-ml fractions were diluted to the concentration of 0.05 mg/ml, their elution volumes did not change and no other oligomerization state formed on the gel filtration column at pH 7.4 (data not shown). These results indicated that M1 can form stable oligomers at neutral pH, and that the oligomerization process is irreversible.

The effect of acidity on oligomerization of M1 was investigated at pH 5.0. M1 purified by nickel affinity chromatography was exchanged into a buffer at pH 5.0. 1.0 mg/ml M1 was loaded onto the gel filtration column, and only the 15.8-ml fraction was observed (Fig. S3A). The fraction was collected and concentrated to higher concentrations by ultrafiltration, but it continued to elute at 15.8 ml on the column (Fig. 1C). The result indicated that the dimeric protein underwent no detectable oligomerization during concentration. Furthermore, it was ascertained that increasing the pH environment of M1 by changing the buffer to pH 7.4 did not change the oligomerization state of M1. M1 dimer alone was observed on the gel filtration column after pH neutralization (Fig. S3C). The pH influence on the high molecular mass oligomers was also examined. We collected the 10.1-ml, 11.2-ml, and 13.5-ml fractions from the gel filtration column at pH 7.4 and exchanged the samples into buffer of pH 5.0. Each sample was loaded on the column at pH 5.0, and all the samples eluted at 15.8 ml. The 15.8-ml fraction generated from acidification of 11.2-ml fraction was shown in Fig. 1D. The result indicated the interactions between dimeric M1 molecules were destroyed by low pH. Taken together, these results indicated that the oligomerization of M1 is dependent on pH, and that the dimeric form of M1 is stable at acidic pH.

The analysis of direct interactions between M1 molecules was carried out by a GST pull-down assay. We found that hisM1 could bind to GST-M1 (Fig. 2B, lane 3), but not to GST alone at pH 7.4 (Fig. 2B, lane 2). The hisM1 was pulled down by GST-M1 at pH 7.4 and was then washed with an acidic buffer (pH 5.0), but no hisM1 was detected by the anti-His antibody (Fig. 2B, lane 4). As M1 forms dimers at pH 5.0, this result indicated that the direct interaction between dimeric M1 could be inhibited by low pH.

### The N-terminal domain is responsible for the pH-dependent oligomerization of M1

To determine the roles of the two domains in M1 oligomerization, we further analyzed the oligomerization behavior of the N-terminal (1–170 aa) and C-terminal (165–252 aa) domains.

Recombinant M1N was constructed with a C-terminal His_6_-tag and expressed in soluble form in *E. coli*. M1N was purified by nickel affinity chromatography and showed a single band of about 18 kDa on Tricine-SDS-PAGE (Fig. S1A, lane 5). Far-UV CD spectroscopy showed that the secondary structure of the recombinant protein was composed of 72% α-helical and 28% random coil, which was consistent with the crystal structure (70% α-helical) (Fig. S1B).

**Figure 3:**
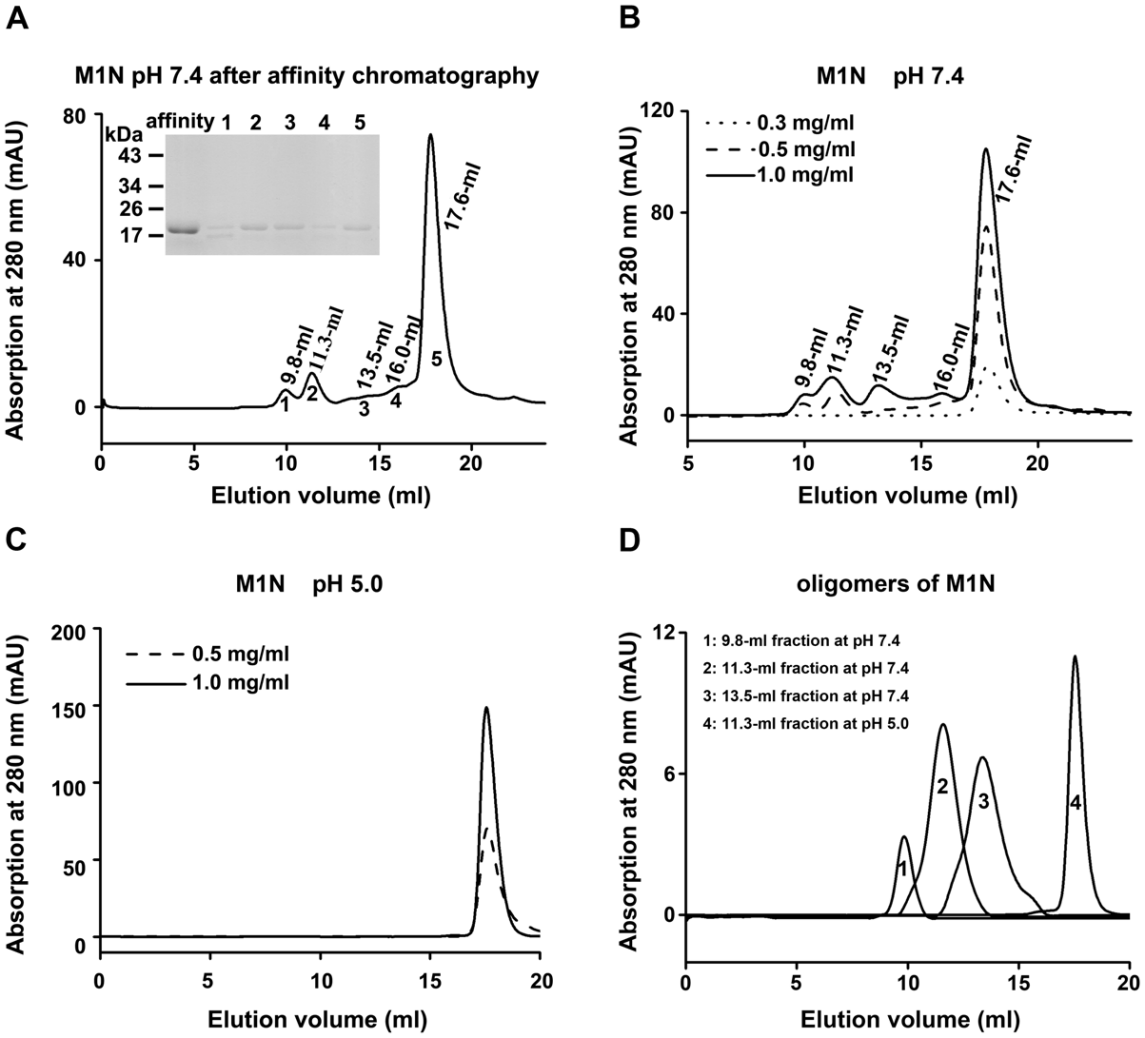
The oligomerization of M1N analyzed by gel filtration. (A) Purified M1N from nickel affinity chromatography yielded five peaks on Superdex 200. The volumes of elution were indicated. The samples from each peak were collected and analyzed by SDS-PAGE. (B) The 17.6-ml fraction (from panel A) was collected, concentrated and re-applied to the column at different concentrations, at pH 7.4. (C) Different concentrations of M1N all eluted as a single peak on the gel filtration column at pH 5.0. (D) Peak 1, 2, 3 correspond to the 9.8-ml, 11.3-ml, and 13.5-ml fractions of M1N re-applied on the gel filtration column at pH 7.4, respectively. Peak 4 corresponds to the 11.3-ml fraction re-applied on gel filtration column at pH 5.0.

M1N purified by affinity chromatography to 0.9 mg/ml was loaded onto the gel filtration column at pH 7.4. The sample yielded five fractions with the elution volumes of 9.8 ml, 11.3 ml, 13.5 ml, 16.0 ml, and 17.6 ml (Fig. 3A). The apparent molecular mass of the 17.6-ml fraction of M1N was estimated to be 19 kDa, which is consistent with monomeric M1N (19.8 kDa) (Fig. S2). Then the protein was concentrated to different concentrations and re-applied to the gel filtration column. Similar to the intact protein, M1N also exhibited a concentration-dependent oligomerization. Only a single 17.6-ml fraction was observed on the gel filtration column when the loaded protein concentration was 0.3 mg/ml. However, the 0.5 mg/ml sample produced five fractions. The elution volumes of the fractions were 9.8 ml, 11.3 ml, 13.5 ml, 16.0 ml, and 17.6 ml, corresponding to the molecular masses of 654 kDa, 436 kDa, 169 kDa, 51 kDa, and 19 kDa, respectively. The proportion of the first four oligomerization fractions also increased with increasing sample concentration (Fig. 3B). When the loaded protein concentration was above 1.0 mg/ml, some of the protein precipitated after incubation at room temperature for 1 h. Also, we collected 9.8-ml, 11.3-ml, and 13.5-ml fractions individually and re-applied each sample to the gel filtration column. The elution volumes of the fractions stayed the same and no other oligomers were observed on the column at pH 7.4 (Fig. 3D), even when the concentrations of the 9.8-ml, 11.3-ml, and 13.5-ml fractions were diluted to lower than 0.1 mg/ml (data not shown). The data clearly suggested that, like the intact M1, the N-terminal domain can form multi-order oligomers at neutral pH, and that the oligomerization process of M1N is also irreversible.

Purified M1N protein from affinity chromatography was exchanged into a buffer of pH 5.0. The protein at the final concentration of 1.5 mg/ml was loaded onto the gel filtration column at pH 5.0. Only one fraction of M1N, which eluted at 17.4 ml, was observed (Fig. S3B). This fraction was collected and concentrated to higher concentration, yet it was still eluted with a symmetric profile at 17.4 ml (Fig. 3C). Like the intact protein, the N-terminal domain purified in acidic pH did not oligomerize after pH neutralization. Only the 17.4-ml fraction appeared on the gel filtration column after the sample buffer was neutralized to pH 7.4 (Fig. S3D). We also individually collected the 9.8-ml, 11.3-ml, and 13.5-ml fractions from the column at pH 7.4, and transferred them into a buffer of pH 5.0. Each sample was loaded on the gel filtration column at pH 5.0, and all the samples eluted at 17.4 ml. The 17.4-ml fraction generated from acidification of 11.3-ml fraction was shown in Fig. 3D. These results indicated that the interactions between M1N were disrupted in an acidic environment, and moreover, demonstrated that the N-terminal domain contributes to the pH-dependent oligomerization characteristic of the intact protein.

**Figure 4:**
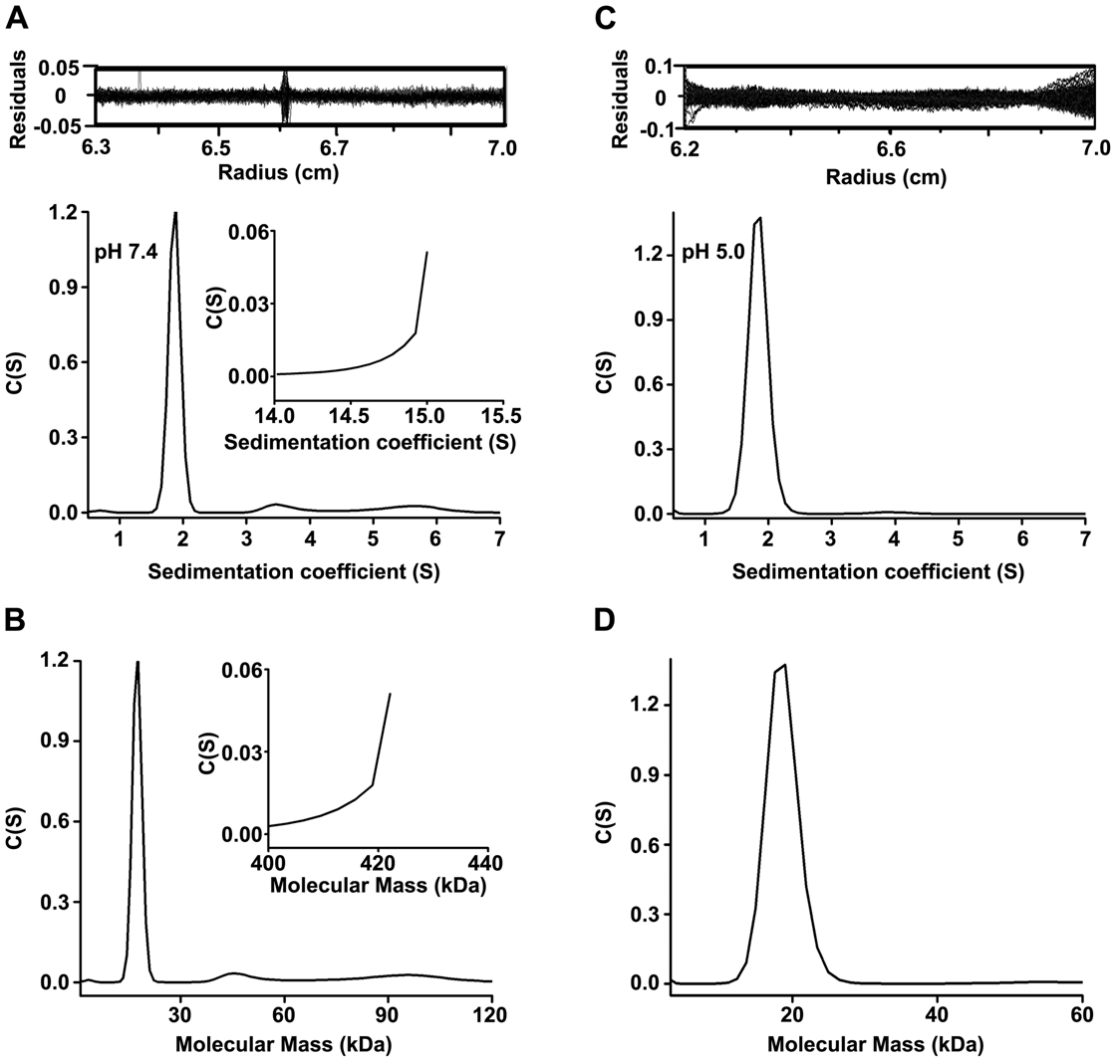
Sedimentation velocity analytical ultracentrifugation determined the oligomerization statue of the N-terminal domain. (A and C) The panels showed the sedimentation coefficient distributions *c(s)* of purified M1N from the 17.6-ml fraction at pH 7.4 (A), and from the 17.4-ml fraction at pH 5.0 (C). The data were acquired at a protein concentration of 1.2 mg/ml. (B and D) The molecular mass distribution derived from *c(s)* data of panel A and C, respectively.

To more accurately observe the oligomerization status of M1N, we also performed sedimentation velocity analytical ultracentrifugation. Unfortunately, due to degradation issues, we could not resolve the sedimentation coefficient distributions (*c(s)*) of intact M1. The M1N samples were collected from a 17.6-ml fraction at pH 7.4 and a 17.4-ml fraction at pH 5.0 from the gel filtration column, and then concentrated to 1.2 mg/ml, respectively. The *c(s)* value was analyzed accordingly (see materials and methods). Sedimentation velocity of M1N at pH 5.0 gave rise to a single peak at 1.8 S (Fig. 4C). This corresponds to a molecular mass of 18.8 kDa (Fig. 4D), which closely agrees with the mass of its monomeric form. At pH 7.4, the data showed the presence of three species. The major species was at 1.8 S, and the sedimentation coefficients of the other two species were 3.5 S and 5.6 S (Fig. 4A), corresponding to molecular masses of 48 kDa and 96 kDa (Fig. 4B). No species higher than 100 kDa were observed. The ascending curve, which appeared at 15.0 S species, indicated that some of the sample might precipitate during centrifugation (Fig. 4A). Taken together, we concluded that the smallest oligomerization state of M1N is a monomer. M1N can form higher molecular mass oligomers at pH 7.4, but it exists only in monomeric form at pH 5.0.

**Figure 5:**
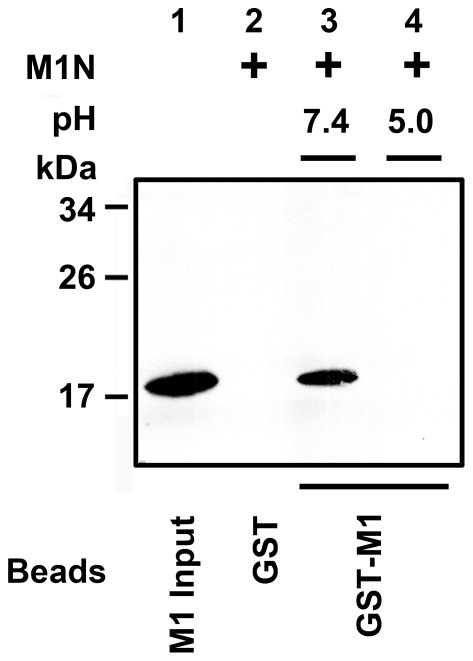
GST pull-down detected the direct interaction between N-terminal domains. Glutathione-Sepharose bound GST-M1 or GST was incubated with M1N in the binding buffer (pH 7.4). After washing extensively with the buffer of different pH (7.4 or 5.0), the M1N bound to the beads was extracted and analyzed by immunoblotting with anti-His antibody.

To test the self-interaction between N-terminal domains in solution, we examined their direct binding activity to a panel of GST fusion proteins. GST-M1 was used to pull down M1N as GST-M1N was expressed in insoluble form in *E. coli*. The possible interaction between M1N and M1C was excluded, as GST-M1 could not pull down M1C. M1N could bind to GST-M1 (Fig. 5A, lane 3), but not to GST alone at pH 7.4 (Fig. 5A, lane 2). The result suggested that N-terminal domains can directly interact with each other at neutral pH. The effect of pH on binding activity was further examined by incubating GST-beads-bound GST-M1 with M1N for 2 hours at pH 7.4, and then washing with acidic buffer (pH 5.0). No M1N was detected by anti-His antibody (Fig. 5A, lane 4). These data indicated that the direct interactions between N-terminal domain were disrupted by low pH and confirmed that oligomerization of N-terminal domain is pH-dependent.

### The C-terminal domain is a stable dimer

**Figure 6:**
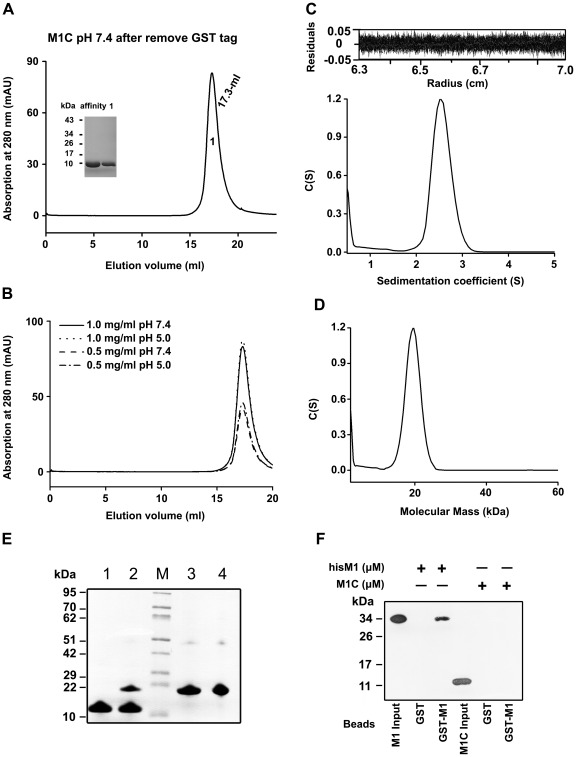
The characterization of oligomerization of C-terminal domain. (A) After affinity chromatography, purified M1C without the N-terminal GST tag (affinity), yielded a single peak (1) on a Superdex 200 column. The samples were also analyzed by Tricine-SDS-PAGE. (B) The 17.3-ml fraction of C-terminal domain from panel A was collected, concentrated and re-applied to gel filtration column. The elution positions of M1C with different loading concentrations and at different pHs are shown. (C) The panel showed the sedimentation coefficient distributions *c(s)* of purified M1C with the concentration of 2.8 mg/ml at pH 7.4. (D) The molecular mass distribution of M1C derived from *c(s)* data of panel C. (E) Cross-linking of M1C. Lane 1 is M1C in the absence of cross-linker, and lane 2 is the cross-linked M1C dimer. Lane 3 and 4 are M1N without/with treated by cross linking agent, respectively. The center lane is the molecular mass marker. (F) Pull-down assay was carried out at pH 7.4. Purified M1C from gel filtration was bound to GST-beads bound GST-M1 in the binding buffer (pH 7.4). The hisM1 bound with GST-M1 was used as positive control. M1C or hisM1 incubated with GST was used as negative control. The bound M1C were examined by anti-M1 mono-antibody.

The C-terminal domain (residues 165–252) of M1 was expressed as a GST-fusion protein. M1C was purified using Glutathione Sepharose 4B resin. After cleavage of the GST tag at the N-terminal by PreScissors protease, the protein migrated as a single band of about 10 kDa in the PAGE (Fig. S1A). We then analyzed the oligomerization of M1C with gel filtration. After the GST tag was removed, M1C was loaded on the gel filtration column at pH 7.4, yielding a single fraction with an elution volume of 17.3 ml (Fig. 6A). That fraction was collected, concentrated and re-applied on the column. Unlike the N-terminal domain, only one fraction of 17.3 ml eluted on the column, independent of pH and concentration variations (Fig. 6B). The apparent molecular mass of the species was calculated to be 25 kDa (Fig. S2), which is between two and three times the theoretical value of monomer (9.7 kDa). Consistent with the gel filtration result, the 17.3-ml fraction existed as one species with the diameter of 2.6 nm in dynamic light scatting (DLS) (Fig. S4). No other oligomers were formed, indicating that the 17.3-ml fraction is stable and that proteins of this fraction do not interact with each other.

Sedimentation velocity analytical ultracentrifugation was also used to analyze the oligomerization states of C-terminal domain from the accurate molecular mass of protein species. M1C existed as a mono-disperse species with a sedimentation velocity value of 2.5 S at pH 7.4 (Fig. 6C). This *c(s)* value corresponds to a molecular mass of 19.6 kDa (Fig. 6D), indicating that M1C exists as a stable dimer in solution. The dimeric form of M1C was further confirmed by cross linking with glutaraldehyde, which resulted in the 17.3-ml fraction forming two bands on Tricine-SDS-PAGE. One band was at the same position as the untreated sample, and the other ran to the position of about 20 kDa, which is about the molecular mass of an M1C dimer (Fig. 6E). The molecular mass of M1C obtained by analytical gel filtration is larger than that obtained by analytical ultracentrifugation, possible due to its molecular shape. Together, these results indicated that there is direct interaction between C-terminal domains, and the C-terminal domain exists in dimeric form.

In the GST pull down assay, no M1C was detected bound to GST-M1 (Fig. 6F). Combined with the results of the pull down assay of N-terminal domain, the data suggested that the N-terminal domain directly binds to the N-terminal domains of other M1 molecules, while the C-terminal domain forms a stable dimer that does not interact with other M1 molecules. As a result, we can conclude that the smallest oligomerization state of M1 is a dimer, and that dimeric M1 could form higher oligomers through direct interactions between N-terminal domains.

**Figure 7:**
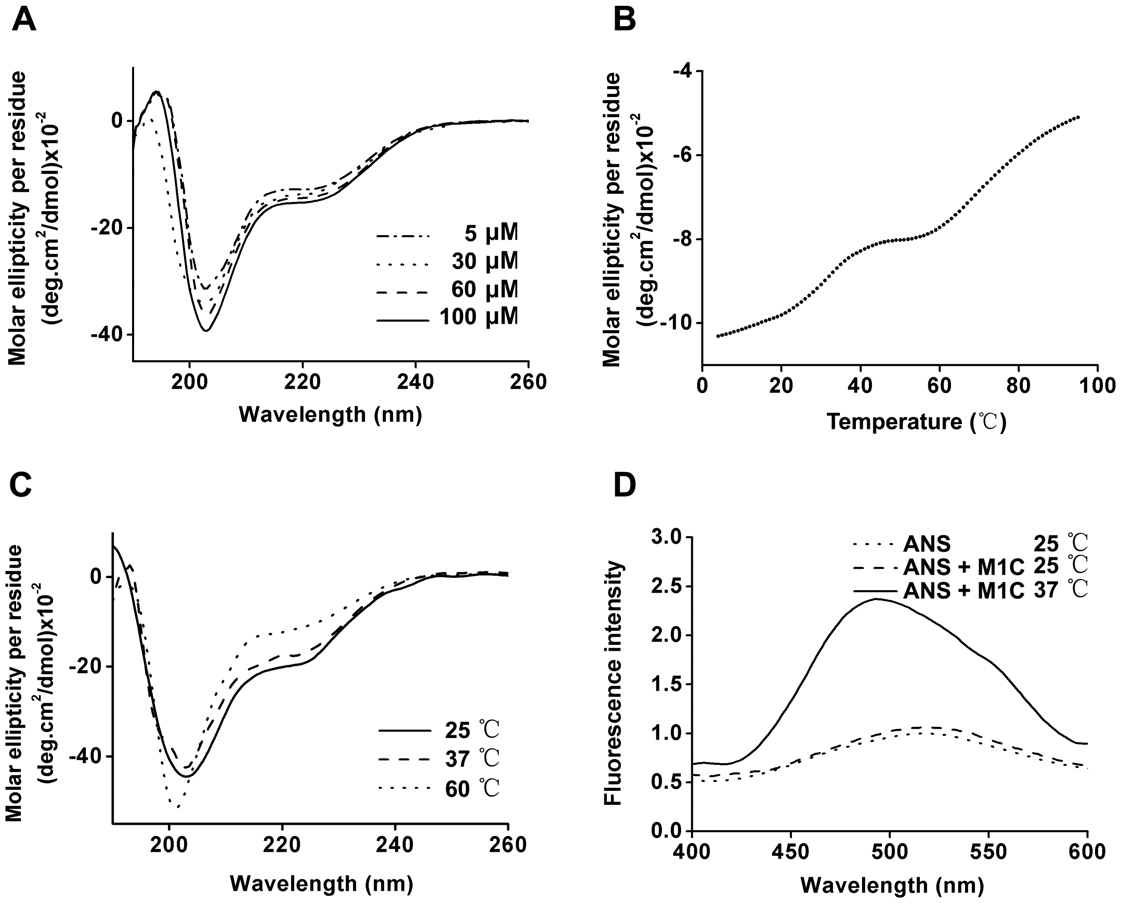
The structural characteristics of M1C analyzed by CD and Fluorescence Spectroscopy. (A) Secondary structure studies on M1C at pH 7.4. The CD spectra of M1C at the concentrations of 5 µM (dash dot line), 30 µM (dot line), 60 µM (dash line), and 100 µM (solid line) are shown. (B) Thermal denaturation of M1C at 60 µM protein concentration was recorded at 222 nm at pH 7.4. (C) Far-UV CD analyses of M1C incubated at room temperature (solid line), at 37°C for 10 min (dash line), and at 60°C for 10 min (dot line) are shown. (D) Fluorescence spectra of ANS binding to M1C at pH 7.4. The fluorescence spectra of ANS (dot line), ANS in presence of M1C at 25°C (dash line) and ANS bound with M1C at 37°C (solid line) are shown.

To further analyze the structural characteristics of the C-terminal domain, several spectroscopy experiments were also performed. Far-UV CD spectra of M1C were collected over a range of protein concentrations at both neutral (Fig. 7A) and acidic pH (data not shown). In all case, nearly identical curves were obtained, indicating the consistency of the secondary structure in this domain. The spectrum of M1C showed a clear minimum at 203 nm and a weak minimum at 222 nm. The minimum at 222 nm was indicative of α-helix content, while the minimum at 203 nm could be the result of a combination of α-helix (208 nm) and random coil structure (200 nm). The exact secondary structure composition of the three proteins was further analyzed by DICHROWEB, with the above data converted into mean molar ellipticity per residue. The results showed that the C-terminal domain is composed of 31% α-helix and 69% random coil. Thermal-induced denaturation of M1C measured by recording ellipticity at 222 nm further revealed the possible heat-induced conformation change. The data showed a three-state transition with the apparent presence of intermediate(s) during denaturation. The two transition points were 32°C and 69°C (Fig. 7B). The content of α-helix was greatly decreased while that of certain random coil structure was induced with the increase of temperature. To validate this, M1C was incubated at two temperature points, and then analyzed by CD (Fig. 7C). At 60°C, the ellipticity value at 222 nm increased, and the other minimum moved from 203 nm to 201 nm, both representing the loss of helix structure. Moreover, the denaturation of M1C was found to be fully reversible, as almost identical CD spectra were obtained for native refolded M1C that were cooled from 90°C to 25°C.

To characterize the hydrophobic cluster of M1C, the transformation of 4,4-Dianilino-1,1-binaphthyl-5,5-disulphonic acid (ANS) fluorescence spectra was investigated. ANS is a fluorescent probe that has been widely used to monitor the hydrophobic exposure of proteins. Fluorescence of ANS is weak in polar solvents, but is enhanced through the association with exposed hydrophobic region of proteins. As shown in Fig. 7D, compared to ANS probe alone, no obvious ANS fluorescence intensity change was observed when adding dimeric M1C, which suggested that M1C has a hydrophobic core and that little hydrophobic area was exposed at 25°C. In contrast, the fluorescence intensity of ANS was slightly enhanced when M1C was heated at 37°C, and its emission maximum was blue-shifted. Therefore, this result also indicated that the hydrophobic core of M1C is exposed to solution at 37°C, which is higher than the first transition point.

## Discussion

Matrix proteins play a crucial role in virus assembly. They can interact with the RNP complex, as well as with the viral membrane. The importance of oligomerization has been observed in a number of viral matrix proteins. Several examples of this include: Nipah virus M, which has a tendency to oligomerize [49], when expressed alone is sufficient to form VLPs [50]; oligomerization of Measles virus M protein can promote the release of VLPs [51]; the tetrameric form of Borna virus M protein is reported to be the basic oligomerization species for the assembly of larger two-dimensional lattices [52]; and oligomerization of Ebola virus VP40 is essential for particle morphogenesis and regulation of viral transcription [53].

In influenza A virus particles, Ruigrok *et al.* reported that M1 is a 6 nm-long rod [7], [41]. It forms an ordered helical layer adjacent to the envelope in the virus particles [42]. Schulze *et al.* estimated that this protein layer was about 60 Å thick [54]. The oligomerization of M1 has been studied from many aspects. M1 oligomers extracted from the virus were found to form flexible ribbons and small coils [41]. Furthermore, when M1 alone was expressed in cells, they found that M1 tends to oligomerize soon after synthesis [48] and produce VLPs.

In our present study, we investigated the oligomerization of M1 *in vitro*. Recombinant M1, expressed in soluble form in *E. coli*, were able to form multi-ordered oligomers at pH 7.4. We determined the smallest oligomerization state of M1 to be a dimer, and we found the oligomerization of M1 to depend on pH. M1 oligomers were able to disassemble to dimeric form in acidic pH. To our knowledge, this is the first report on the detection of M1 oligomers *in vitro*. Previous studies showed that M1 extracted from the virus at acidic pH could not oligomerize after pH neutralization [15]. We also identified recombinant M1 purified *in vitro* at acidic pH that could also not oligomerize after neutralizing the buffer pH. In more in-depth studies, we dissected the contributions of the N- and C-terminal domains to the oligomerization of M1 in detail.

The crystal structure of N-terminal domain solved by Sha and Harris showed that this domain is in dimeric form [31], [32]. But Arz *et al.* reported that the N-terminal domain is a monomer in solution [30]. In our study, we identified that the smallest oligomerization state of the N-terminal domain is a monomer. Interaction interfaces between N-terminal domains were observed in the crystal structure, and Noton *et al.* reported that the middle region dominates the oligomerization of M1 [10]. Here, we found that N-terminal domain can form multi-order oligomers at pH 7.4, and confirmed the direct interaction between N-terminal domains. The results indicated that self-oligomerization with the N-terminal domain is important in the oligomerization of M1. Further studies showed the oligomerization of N-terminal domain is pH-dependent. The interaction between N-terminal domains is disrupted at pH 5.0, causing the oligomers of M1N dissociate to monomeric form. Moreover, our findings indicated that the N-terminal domain contributes to the pH-dependence of oligomerization in the intact protein. During the uncoating process of virus infection, H^+^ effluxes from endosomes into virus particles and acidifies the interior of the virion. Calder *et al.* found that matrix layer could disassemble at low pH [42]. The recent studies further showed the matrix layer would disappear when the virions were incubated at pH 4.9 for 30 min [47]. Our findings suggest that dissociation of M1 oligomers may result from the disruption of interactions in N-terminal domain at low pH. Our data also showed that the N-terminal domain purified at acidic pH could not oligomerize after pH neutralization, which may cause the same phenomenon observed in M1. This result could be related to the release of vRNP into the nucleus from M1-vRNP complexes, during which the interaction between M1 and vRNP is destroyed under acidification [9]. As low pH induces an irreversible conformation change of M1, it would be unable to re-associate with vRNP in the cytoplasm [55].

The atomic structure of C-terminal domain has not been resolved yet. The results of tritium-bombardment experiments suggest that the C-terminal domain consists largely of α-helix [33], [34], [35]. Arzt *et al* calculated the C-terminal domain to have a structure consisting of 38% α-helix and considered that C-terminal domain contributes more size to monomeric intact protein than its molecular weight [30]. Our DLS data support the view about the size of the C-terminal domain. The diameter of the C-terminal domain is 2.6 nm, larger than the global protein with a molecular mass of 20 kDa (about 2.1 nm). We directly measured the α-helix content in the C-terminal domain to be 31%, and examined the structural plasticity by several methods. It has been proposed that the weakly structured C-terminal domain contributes more to the flexibility of M1, and considerably unstructured regions enable C-terminal domain to interact with some components of the host cell [35], [36]. On the other hand, previous research has identified the important role of the C-terminal domain in virus assembly. Studies by Noton *et al.* showed that the N-terminal domain alone is not sufficient for virion assembly [10]. M1 SUMOylated at K242 has also been identified to be involved in the process of virus assembly [56]. The mechanisms of how the C-terminal domain participates in the oligomerization of M1 reported by Noton *et al.* [10] and Ruigrok *et al.* were inconsistent. Our results disagree with either of them. In our study, we have demonstrated that the C-terminal domain is a stable dimer independent of pH and protein concentration. There is no direct interaction between dimeric forms of C-terminal domains. Therefore, it is reasonable to infer that the smallest oligomerization state of M1 is dimer. Our results also demonstrated that the C-terminal domain does not interact with the N-terminal domain. Together with the result that the interaction between N-terminal domains is disrupted at acidic pH, leading the N-terminal domain completely dissociates into a monomer, we conclude that M1 forms a stable dimer at low pH.

In summary, we investigated the oligomerization of M1 of influenza A virus, and found that the N-terminal domain contributes to the pH-dependent oligomerization characteristic of M1. The C-terminal domain is reported for the first time to be a stable dimer. Both N- and C-terminal domains are involved in the oligomerization of M1, which is important in the influenza A virus life cycle. The present data may provide us with new insights into the mechanism of the formation of matrix layer, and help us to better understand the dissociation of the matrix layer of influenza A virus in the uncoating process.

## Materials and Methods

### Reagents, *Escherichia coli* strains and antibodies

*Escherichia coli* (*E. coli*) strains DH5α and BL21 (DE3) pLysS were obtained from Novagen. High purity chemicals were purchased from Sigma-Aldrich. Other suppliers were: DNA polymerase, T4 DNA ligase and restriction enzymes (New England Biolabs); pET30a vector (Novagen); pGEX-6p-1 vector (GE Healthcare); Yest Extract and Tryptone needed for Luria-Bertani (LB) media (Merck); All kits and devices for protein purification (GE Healthcare); His probe antibody and HRP labeled goat anti-mouse antibody (Santa Cruz Biotechnology); Chemiluminescent for Western blot (Pierce). Mouse anti-M1 monoclonal antibody and rabbit anti-M1 polyclonal antibody were prepared as described previously [57].

### Recombinant plasmids construction

Influenza A virus A/chicken/Jiangsu/1/1998 (H9N2) M1 genes encoding full-length M1 (252 residues) and an N-terminal fragment (1–170 aa) were amplified by PCR. The amplified DNAs were purified and digested with *Nde*I/*Xho*I enzymes. Then cloned into the corresponding sites of pET30a vector (Novagen) with a His_6_-tag coding sequence (LEHHHHHH) fused at the carboxyl terminus. Recombinant plasmids pET30a-M1 and pET30a-M1N were obtained. The purified PCR product of M1C fragment (165–252 aa) was amplified and cloned into pGEX-6p-1 vector using *BamH*I and *Xho*I restriction sites to obtain recombinant plasmid pGEX-6P-1-M1C. Plasmids pGEX-6p-1-GST-M1 and pET30a-hisM1 were also constructed following the similar method. All the above constructs were transformed into *E. coli* strain DH5α and inserted genes were confirmed by DNA sequencing.

### Protein expression and purification

Plasmids pET30a-M1, pET30a-M1N, pET30a-hisM1 and pGEX-6P-1-M1C were transformed into *E. coli* strain BL21 (DE3) and expressed M1, M1N, hisM1 as His_6_-tag proteins, while M1C as glutathione S-transferase (GST) fusion protein. Cells were grown at 37°C in LB medium containing ampicillin, 100 µg/ml; kanamycin, 50 µg/ml to an OD_600_ of 0.8–1.0. The culture was harvested by centrifugation after induction with 0.5 mM IPTG (isopropyl-1-thio-β-D-galactopyanoside) at 16°C for 11 h. Then the cells were resuspended in binding buffer (His_6_-tagged proteins: 20 mM Tris, 500 mM NaCl, 20 mM imidazole, 0.1 mM PMSF, pH 7.9; GST-tagged protein: Phosphate buffer saline (PBS), 0.1 mM PMSF, pH 7.4) and lysed by sonication. Insoluble materials were removed by centrifugation at 13,000 g at 4°C for 20 min. Affinity chromatography was used as the first step of the purification. Supernatant of M1 and M1N were flowed through a chelating Sepharose Fast Flow Column, and the bonded proteins were eluted with elution buffer (20 mM Tris, 500 mM NaCl, 500 mM imidazole pH 7.9). Supernatant of M1C was applied to a column contained affinity resin (Glutathione Sepharose 4B) and eluted with elution buffer (50 mM Tris, 50 mM reduced glutathione, pH 8.0). It was then changed to Cleavage Buffer (50 mM Tris, 150 mM NaCl, 1 mM EDTA, 1 mM DTT, pH 7.0), and PreScission Protease and GST-tag were eliminated by passing through the affinity column twice at 4°C followed by further purification by gel filtration using A Superdex 200™ 10/30 column (10 mm×300 mm) on an ÄKTA fast protein liquid chromatography system (FPLC).

### Analytical gel filtration analysis

Affinity purified proteins were fractionated by analytical gel filtration on a Superdex™ 200 HR 10/30 column (10 mm×300 mm). The column was equilibrated with two column of elution buffer before experiment. The process was executed at a flow rate of 0.5 ml/min at constant room temperature of 25°C. Absorbance was monitored at 280 nm, and elution volumes were determined from UV chromatogram. The column was calibrated with the following standard globular proteins: Ribonuclease A (13.7 kDa, 18.8 ml), Cyclophilin A (18.0 kDa, 18.0 ml), Rabbit Action Ovabumin (43 kDa, 16.2 ml), bovine albumin V (68.0 kDa, 15.1 ml) and Aldolase (158 kDa, 13.3 ml). The void volume of column was determined by Blue dextran 2000. The partition coefficient, Kav, was calculated from the elution volume of the sample. A standard calibration curve was obtained by plotting the ratio (V_e_−V_0_)/(V_t_−V_0_) against the logarithm of molecular mass (V_e_ is elution volume, V_0_ is void volume and V_t_ is total bed volume) by UNICORN™ software. The apparent molecular mass of the sample can be estimated based on the acquired straight line.

The determination of proteins oligomerization was performed by injection of purified proteins from affinity chromatography onto the gel filtration column at pH 7.4 and pH 5.0. The largest elution volume fraction of M1 or M1N or M1C on gel filtration column was collected and concentrated to a series of concentrations in 20 mM Tris, 150 mM NaCl, pH 7.4 or 20 mM sodium acetate, 150 mM NaCl, pH 5.0 by Ultrafiltration. Then 500 µl each sample was load onto the column and eluted with the same buffer to protein solutions.

### Analytical ultracentrifugation

Sedimentation velocity was performed in a Beckman-Coulter XL-I analytical ultracentrifuge. Sample (400 µl) and buffer (400 µl) solutions were loaded into the double sector centerpiece separately, and equipped with a four-cell An-60 Tirotor. 1.2 mg/ml M1N was equilibrated in 20 mM Tris, 150 mM NaCl pH 7.4 and 1.2 mg/ml M1N in 20 mM sodium acetate, 150 mM NaCl, pH 5.0. 2.8 mg/ml M1C was equilibrated with 20 mM Tris, 150 mM NaCl, pH 7.4. Experiments were carried out with the rotor speed of 50,000 rpm at 20°C. Scans were monitored at 280 nm with a radial increment step size of 0.003 cm. Differential sedimentation coefficient distributions, *c(s)*, were calculated by least squares boundary modeling of sedimentation velocity data using the *c(s)* method, which as an implement of SEDFIT program on www.analyticalultracentrifugation.com/download.htm [58]. The values of apparent sedimentation coefficients (s-values) were corrected to standard conditions (water, 20°C, and infinite dilution) using SEDNTERP program (from www.jphilo.mailway.com/download.htm.) [59], to get the corresponding s-standard value (s_20, *w*_). The frictional coefficients (*f/f0*) and axial ratios (*a/b*) were calculated by using the vbar method in SEDNTERP program, assuming a prolate ellipsoid model. Sedimentation coefficient distributions was transformed to a molar mass distributions based on the frictional ratio [60].

### GST pull-down assays

GST pull-down assay was carried out to detect the interactions in M1 molecules. Expression and purification of hisM1, M1N, and M1C used in the experiment were followed with the procedure described above, and purified proteins were exchanged into binding buffer (20 mM Hepes, 150 mM NaCl, 1 mM EDTA, 1% NP40, pH 7.4). The GST-M1 or the control GST protein was expressed in *E. coli* BL21. Cells were collected and followed by sonication. The cell lysates were centrifuged at 13,000 g for 15 min at 4°C. The supernatants were applied to a column containing Glutathione Sepharose 4B binding for 1 h at 4°C [61]. The column was washed with 20 column volumes of PBS. An equal amount of either GST-M1 or GST bound to affinity resin was mixed with hisM1 or M1N or M1C in 500 µl binding buffer, and then incubated for 2 h at 4°C. The resin was washed six times with washing buffer (300 mM NaCl added in binding buffer,). The quality of proteins used in the assays as following: 10 µg GST-M1 mixed with 20 µg M1N or M1C or hisM1, 10 µg GST mixed with 20 µg M1N or M1C as negative control. Pull down assay was also performed to analyze the effect of pH on the binding activity. 10 µg GST-M1 or GST bound to resin was mixed with 20 µg hisM1 or 20 µg M1N in binding buffer and was washed four times with washing buffer. Then the resin was washed six times in acidic environment (20 mM NaH_2_PO_4_, 450 mM NaCl, pH 5.0). Proteins bound to the resin were recovered by boiling for 10 min in presence of 2× SDS loading buffer, and subjected to autoradiography. Proteins were then detected by Western blot with an anti-His-tag monoclonal antibody for hisM1, M1N and an anti-M1 monoclonal antibody for M1C.

### Glutaraldehyde Cross-linking

Protein cross-linking was performed at room temperature. The samples were cross-linked with 0.2% (v/v) glutaraldehyde in the reaction buffer (20 mM sodium phosphate, 100 mM NaCl, pH 8.0). The reaction mixture was incubated for 5 minutes, and then the reaction was quenched by adding Tris (1 M, pH 7.5) to a final concentration of 50 mM. Proteins were analyzed by Tricine-SDS-PAGE

### CD spectroscopy

CD spectra at far-UV (190–260 nm) were performed on a Jasco810 spectropolarimeter at room temperature (25°C). A 1 mm quartz cell was used for far-UV CD spectra. M1 and M1N, which were concentrated to 30 µM by Ultra filtration, were recorded in 50 mM sodium phosphate, pH 7.4. Spectra of 5 µM, 30 µM, 60 µM and 100 µM M1C were measured in 50 mM sodium phosphate, pH 7.4 and 50 mM NaH_2_PO_4_, pH 5.0. The spectra were collected at a scanning rate of 1 nm/s with intervals of 0.5 nm. The data were obtained by averaging five repeat scans and corrected by subtraction of the buffer absorption. The resulting spectra expressed as mean molar ellipticity per residue. The secondary structure content was analyzed by DICHROWEB.

Thermal denaturation of M1C was monitored by far-UV CD spectra at 222 nm in 1 mm path length cell. 30 µM M1C in 50 mM sodium phosphate, pH 7.4 was heated from 4°C to 96°C at the scan rate of 0.5°C/min. The sample was cooled down to room temperature immediately when one course complete. The far-UV CD spectrum of renatured protein was compared with that of before heating. The final data of thermal denaturation was averaged by thrice scans.

### DLS analysis

The degree of compactness and particle hydrodynamic diameter of M1C was examined by dynamic Light Scattering DLS. M1C got from on gel filtration column was concentrated to the optimal reactive concentration of 1.0 mg/ml in 20 mM Tris (pH 7.4), and then the measurement was performed at 25°C.

### Fluorescence Spectroscopy

Fluorescence emission spectra were recorded at room temperature by a HITACHI F-4500 fluorescence spectrophotometer in 1 cm quartz cuvette. ANS binding was detected by fluorescence spectra at an excitation wavelength of 380 nm. Emission spectra were measured from 400 nm to 600 nm with the molar ratio ANS/M1C 100∶1. Each spectrum was the average of three emission scans.

## Supporting Information

Figure S1 **Expression, purification of M1, M1N, M1C from** ***E. coli*** **and secondary structure analysis.** (A) M1, M1N and M1C produced in *E. coli* were purified by affinity chromatography. The purified proteins were analyzed by Tricine-SDS-PAGE. Lane M, the molecular mass marker; lane 1, total cell lysate after induction of pET30a; lane 2, total cell lysate after induction of pET30a-M1 for 11 h at 16°C; lane 3, purified M1 from nickel affinity chromatography; lane 4, total cell lysate after pET30a-M1N was induced for 12 h; lane 5, purified M1N from nickel affinity chromatography; lane 6, total cell lysate of pGEX-6p-1-M1C prior to induction; lane 7, total cell lysate of pGEX-6p-1-M1C after induction for 12 h at 16°C; lane 8, GST-M1C eluted from Glutathione Sepharose 4B; lane 9, M1C yielded by PreScission protease cleavage of GST-M1C. (B) Far-UV CD analyses of M1and M1N at the concentration of 30 µM are shown.
(TIF)

Figure S2 **Molecular mass estimation using UNICORN Software.** The apparent molecular masses of the 15.8-ml fraction of M1, the 17.6-ml fraction of M1N and the 17.3-ml of M1C on analytical gel filtration column at pH 7.4 were analyzed using UNICORN Software Analysis Module. The column was calibrated by a series of standard global proteins, and the correlation of Log (Molecular weight) and Kav was plotted. The apparent molecular masses indicated by arrows were calculated from the straight line.
(TIF)

Figure S3 G**el filtration analyses of neutralized proteins obtained from acidic pH.** (A and B) Purified M1 (A) and M1N (B) from nickel affinity chromatography were concentrated to 0.7 and 0.6 mg/ml respectively, and loaded on a Superdex 200 column. Purified proteins from the affinity chromatography and gel filtration were analyzed on SDS-PAGE. (C and D) M1 (C) and M1N (D) were purified from gel filtration chromatography at pH 5.0 (solid line), and neutralized into a buffer of pH 7.4, concentrated and reapplied to the column at pH 7.4 (dot line).
(TIF)

Figure S4 **The molecular size of M1C measured by DLS.** The degree of compactness and particle hydrodynamic diameter of M1C were examined by DLS. Essentially 100% of the scattering mass was attributed to a single species of M1C.
(TIF)
