# Supplementary figures and images for: Dissection of Influenza A Virus M1 Protein: pH-Dependent Oligomerization of N-Terminal Domain and Dimerization of C-Terminal Domain
Source: PLoS One. 2012 May 24;7(5):e37786. doi: 10.1371/journal.pone.0037786 (PMC3360003; doi:10.1371/journal.pone.0037786)

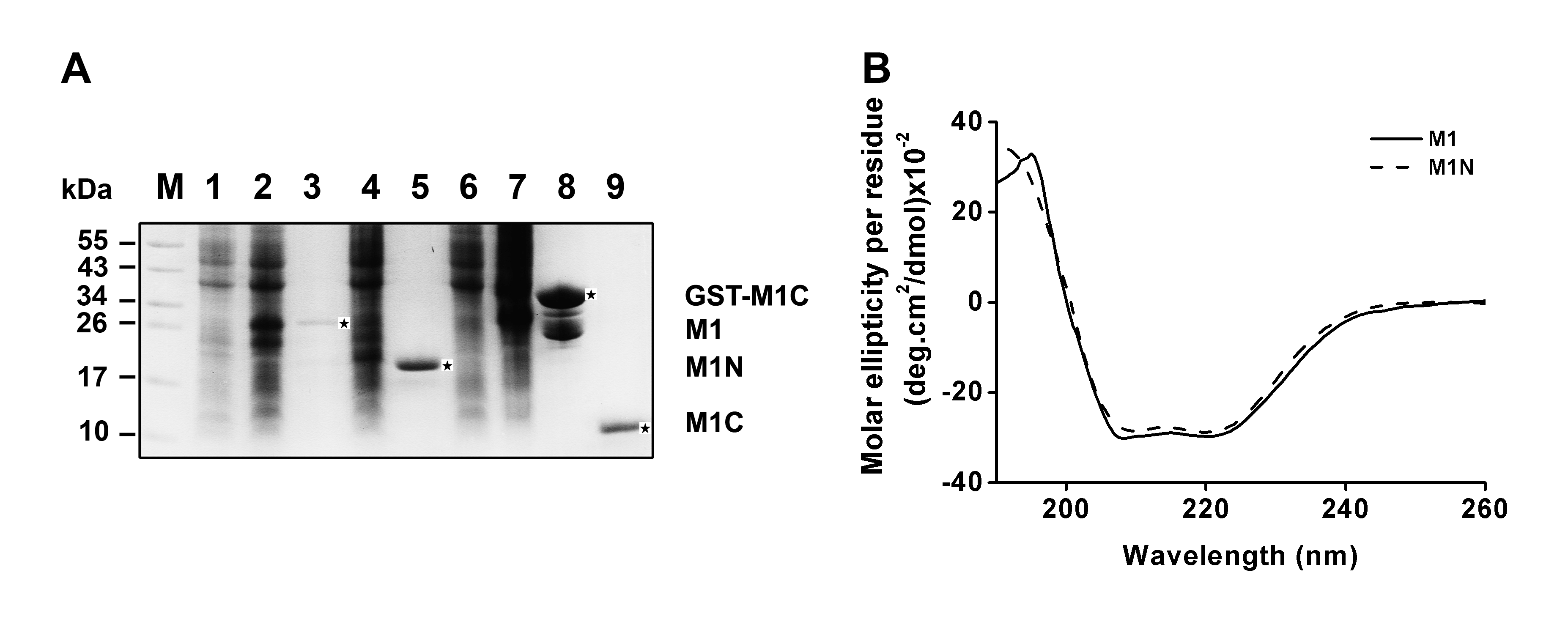

Supplement: Figure S1 — Expression, purification of M1, M1N, M1C from E. coli and secondary structure analysis. (A) M1, M1N and M1C produced in E. coli were purified by affinity chromatography. The purified proteins were analyzed by Tricine-SDS-PAGE. Lane M, the molecular mass marker; lane 1, total cell lysate after induction of pET30a; lane 2, total cell lysate after induction of pET30a-M1 for 11 h at 16°C; lane 3, purified M1 from nickel affinity chromatography; lane 4, total cell lysate after pET30a-M1N was induced for 12 h; lane 5, purified M1N from nickel affinity chromatography; lane 6, total cell lysate of pGEX-6p-1-M1C prior to induction; lane 7, total cell lysate of pGEX-6p-1-M1C after induction for 12 h at 16°C; lane 8, GST-M1C eluted from Glutathione Sepharose 4B; lane 9, M1C yielded by PreScission protease cleavage of GST-M1C. (B) Far-UV CD analyses of M1and M1N at the concentration of 30 µM are shown. (TIF) [file pone.0037786.s001.tif]

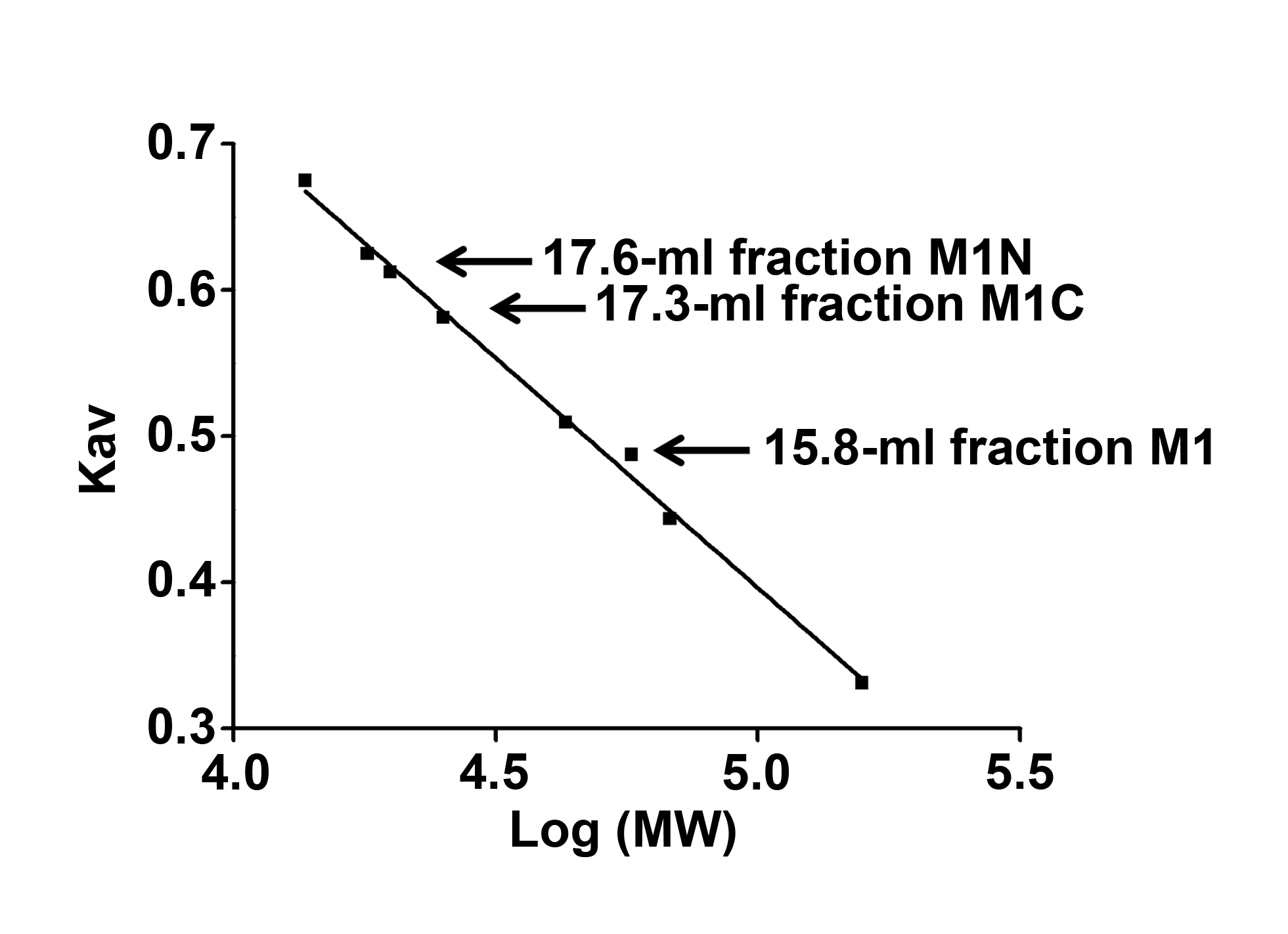

Supplement: Figure S2 — Molecular mass estimation using UNICORN Software. The apparent molecular masses of the 15.8-ml fraction of M1, the 17.6-ml fraction of M1N and the 17.3-ml of M1C on analytical gel filtration column at pH 7.4 were analyzed using UNICORN Software Analysis Module. The column was calibrated by a series of standard global proteins, and the correlation of Log (Molecular weight) and Kav was plotted. The apparent molecular masses indicated by arrows were calculated from the straight line. (TIF) [file pone.0037786.s002.tif]

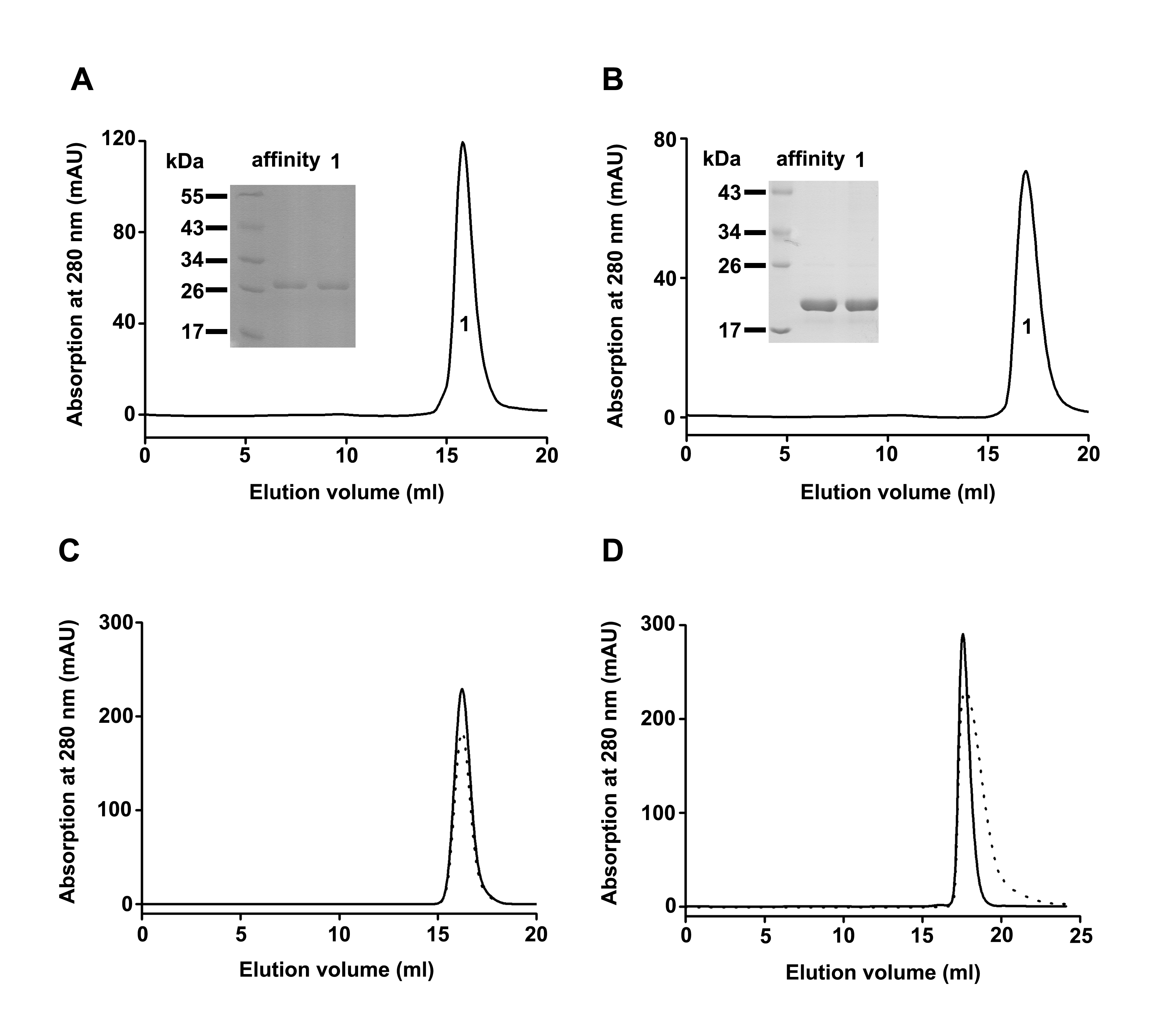

Supplement: Figure S3 — Gel filtration analyses of neutralized proteins obtained from acidic pH. (A and B) Purified M1 (A) and M1N (B) from nickel affinity chromatography were concentrated to 0.7 and 0.6 mg/ml respectively, and loaded on a Superdex 200 column. Purified proteins from the affinity chromatography and gel filtration were analyzed on SDS-PAGE. (C and D) M1 (C) and M1N (D) were purified from gel filtration chromatography at pH 5.0 (solid line), and neutralized into a buffer of pH 7.4, concentrated and reapplied to the column at pH 7.4 (dot line). (TIF) [file pone.0037786.s003.tif]

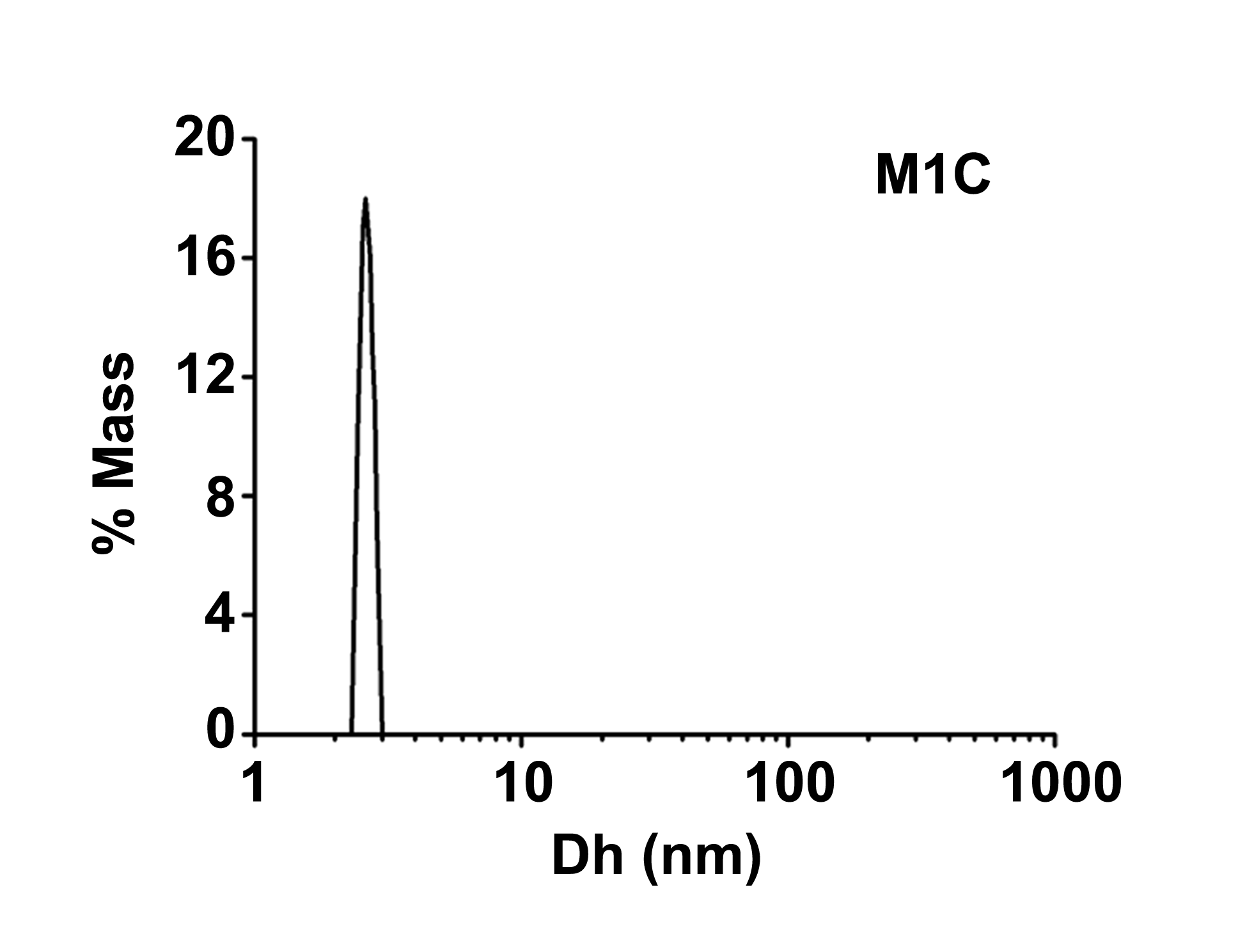

Supplement: Figure S4 — The molecular size of M1C measured by DLS. The degree of compactness and particle hydrodynamic diameter of M1C were examined by DLS. Essentially 100% of the scattering mass was attributed to a single species of M1C. (TIF) [file pone.0037786.s004.tif]
